# The relationship between virulence and drug resistance genes in *Pseudomonas aeruginosa* and antibiotic resistance: a targeted next-generation sequencing approach

**DOI:** 10.3389/fcimb.2025.1563741

**Published:** 2025-05-26

**Authors:** Huanhuan Wei, Yuanyong Fu, Weijuan Qin, Zhengyi Liang, Liyan Zhou, Jingwei Gao, Shiyi He, Xiaoning Wu

**Affiliations:** ^1^ Department of Medical Laboratory, The Second Affiliated Hospital of Guangxi Medical University, Nanning, China; ^2^ Department of Laboratory Medicine, The People’s Hospital of Guangxi Zhuang Autonomous Region (Guangxi Academy of Medical Sciences), Nanning, China

**Keywords:** *Pseudomonas aeruginosa*, resistance genes, virulence genes, targeted next-generation sequencing, multidrug-resistant, carbapenem-resistant *Pseudomonas aeruginosa*

## Abstract

**Objective:**

*Pseudomonas aeruginosa* is an opportunistic pathogen responsible for nosocomial infections in critically ill and immunocompromised patients. To elucidate genomic-phenotypic-clinical correlations in *P. aeruginosa* infections, this study integrates targeted next-generation sequencing (tNGS) with conventional diagnostics.

**Methods:**

A retrospective study was conducted between September, 2023 and December, 2024 at The Second Affiliated Hospital of Guangxi Medical University on patient specimens that were subjected to both conventional culture and tNGS testing. Only a single Gram-negative bacterium, *P. aeruginosa*, was detected by tNGS, and these isolates were subsequently analyzed in order to compare their genomic profiling (virulence/resistance genes), antimicrobial susceptibility testing (AST), and clinical characteristics.

**Results:**

A total of 105 samples were included in the study. Exoenzyme Y gene (*exoY*) and O-antigen polymerase gene (*wzy*) were detected in 77.1% and 12.4% of the samples, respectively. Meanwhile, aminoglycoside acetyltransferase gene (*aac(6’)aac(3’)*), aminoglycoside resistance methyltransferase gene (*armA*), and chloram phenicol resistance genes (*cmlA*) were detected in 6.7%, 4.8%, and 12.4% of the samples, respectively. *P. aeruginosa* isolates detected the *exoY* gene exhibit a higher level of drug resistance, particularly to cefepime (32.1% vs 4.2%, *p*<0.05) and piperacillin-tazobactam (33.3% vs 8.3%, *p*<0.05). *aac(6’)aac(3’)* and *armA* genes were statistically associated with tobramycin resistance (*p*<0.05). The use of antibiotics before hospitalization (within 90 days), hospital-acquired infections, ICU admission, as well as pre-*P. aeruginosa* detection interventions including invasive procedures, catheterization, mechanical ventilation, duration of antibiotic use time≥14 days, and antibiotic use type ≥3 types (*p*<0.05) is associated with multidrug-resistant (MDR) and carbapenem-resistant *P. aeruginosa* (CRPA) infections (*p*<0.05). Furthermore, the detection of MDR and CRPA strains appears to lead to an increase in the duration and variety of antibiotic use, as well as prolonged of hospital stay (*p*<0.05).

**Conclusion:**

Our study highlights the importance of integrating tNGS results, which provide insights into pathogen identification, resistance, and virulence genes, with phenotypic and clinical data in order to enhance the accuracy of diagnosis and guide treatment strategies.

## Introduction

*Pseudomonas aeruginosa* is a Gram-negative bacterium commonly found in many different environments, including soil, water, plants, and animals, as part of the natural bacterial microbiota, but it is also known to be an opportunistic pathogen that is a frequent cause of nosocomial infections, such as pneumonia, surgical site infections, urinary tract infections, and bacteremia (Sommer et al., 2020). Apart from its high intrinsic resistance, *P. aeruginosa* exhibits an exceptional ability to acquire resistance to almost all available antibiotics (Botelho et al., 2019). Infections caused by multidrug-resistant (MDR) or extensively drug-resistant (XDR) *P. aeruginosa* are linked to a significant increase in morbidity and mortality (Del Barrio-Tofiño et al., 2020; Reynolds and Kollef, 2021). Carbapenems are the preferred treatment for severe *P. aeruginosa* infections, but resistance to this class of antibiotics has recently emerged as well in the eponymous carbapenem-resistant *P. aeruginosa* (CRPA) (Teo et al., 2021).

The infection potential of *P. aeruginosa* is partly attributable to the presence of virulence factors and partly to its ability to metabolize a wide range of antibiotics, which is encoded by genes that are collectively organized within genomic islands, making it harder for any single antibiotic to be effective (Botelho et al., 2019). *P. aeruginosa* utilizes a type III secretion system (T3SS) to inject cytotoxic effector proteins into host cells, and the promiscuous nucleotidyl cyclase, exoenzyme Y (ExoY), is one of the most common effectors found in clinical *P. aeruginosa* isolates (Mancl et al., 2020). Phosphorylating (APH), adenylylating (ANT), and acetylating (AAC) enzymes compose the three classes of aminoglycoside-modifying enzymes. The acetyltransferases are a particularly important class of resistance enzymes because of their ability to inactivate many of the medically useful aminoglycosides, such as gentamicin, tobramycin, amikacin, and netilmicin (Schwocho et al., 1995). Both ArmA and RmtB, which belong to the 16S rRNA methyltransferase (16S RMTase), have been shown to contribute to aminoglycoside resistance (O’Hara et al., 2013).

In terms of methods of genomic analysis, metagenomic next-generation sequencing (mNGS) work-flows sequence as much DNA and/or RNA as possible in a given sample, whereas targeted next-generation sequencing (tNGS) workflows enrich specific genetic targets for sequencing and therefore have the advantage of enriching genetic targets for specific pathogens or pathogen groups, as well as other genes of interest (Gaston et al., 2022; Ma et al., 2024). Additionally, tNGS can detect drug resistance and virulence genes. However, the clinical application of pathogens identified through tNGS, along with their associated drug resistance and virulence genes, remains inadequately studied. This article therefore compiles data on sequence counts and resistance and virulence genes of *P. aeruginosa* from tNGS tests conducted at The Second Affiliated Hospital of Guangxi Medical University. By integrating antimicrobial susceptibility test results with patient clinical characteristics, we sought to examine the relationship between the virulence and resistance genes of *P. aeruginosa* and antibiotic resistance as thoroughly as possible.

## Materials and methods

### Patient and sample collection

A total of 105 patients admitted to The Second Affiliated Hospital of Guangxi Medical University from September, 2023 to December, 2024 who had *P. aeruginosa* detected in their tNGS results were retrospectively enrolled. The inclusion criteria were as follows: (1) *P. aeruginosa* being the sole Gram-negative bacterium detected with tNGS; (2) Specimens being subjected to both conventional culture and tNGS in parallel; and (3) complete clinical data. The exclusion criterion was: (1) Patients from potential transplant units.

### Targeted next-generation sequencing

The tNGS assay employs 2,320 specific primers to detect 276 pathogens. Within the pathogen detection module, *Mycobacterium tuberculosis* complex receives the highest primer allocation (n=73), while *P. aeruginosa* is targeted by seven specifically configured primer pairs. Additionally, 269 primers were designed for detecting resistance and virulence genes. The primers related to *P. aeruginosa* are shown in **Table 1**. Application and practice of tNGS were followed the guidelines of expert consensus (Healthcare, 2024).

**Table 1:** Primers used in the present study related to *P. aeruginosa*.

Target gene	Primer sequence (5’-3’)
*aac(3’)*	F-GAGCAGCCGCGTAGTGAGAT
	R-CGCGTTGGCCTCATGCTT
*aac(6’)*	F-ATTCGTATATTAGTGATGAATTATCTATACTAGGTT
	R-ACTGGCAATATCTCGTTTTAACAAATTT
*wzy*	F-AAACCAAGGAAGGCGAATGTTAGTG
	R-TGCCCAGCAAAGTCAAAGGAAAAAT
*exoY*	F-CGGAAATACGCAAGCTGAACCTG
	R-GAAATTCCGCACCAGCTCCG
*armA*	F-TGATGTTGTTAAGAAGATACTTGAATCAAAA
	R-ACCCCCATATTTGATGCAATTTCTTT
*wzz*	F-TCGATTCTCAAGATCTCCGTGAACA
	R-TTACGATTCGCTCTATTAACGCTCT
*cmlA*	F-TACTTCTGCTTGGGCGGTGTACT
	R-GACTGTTGCAAGGGTCAAACAGTAC
*ant*	F-CGTGGAGCGATATCGATTTCGAT
	R-CCGAGATATGCACCAGCCG
*rmt*	F-GGAAGAGGCCTCTGGTTTGAAAATT
	R-TCTGACCCAACAAGATCATTCTCGA
*P. aeruginosa*, N15_031	F-TCGGTACCCTGTCGATCCTG
	R-TTGGCTTCGGAATGACGTTC
*P. aeruginosa*, N15_031_S2	F-GCCGGTGTAATTGCGTATGA
	R-AAGTCTGGCTTGCGCTTCA
*P. aeruginosa*, N15_031_S3	F-CCCGTTACAGACGGCTTACA
	R-CTCACGCATGGATACCGTC
*P. aeruginosa*, N15_031_S4	F-GGGTATTTCTGGCCGAACATC
	R-TACAGTGGAAGCAGCTCTG
*P. aeruginosa*, N15_031_S5	F-AGACCGTGACTACCTCATTGC
	R-GCTTCCCTGTTGCATGTGA
*P. aeruginosa*, N15_031_S6	F-CTATTCGACTACTTCAAACTACGATCCT
	R-GTTCTTCAGCAGGATGACAAACTG
*P. aeruginosa*, N15_031_S7	F-TGACGAGCAAGCGCTAAACTTA
	R-AGCAAAGGCGAGGATCGAG

tNGS detection was completed by the Department of Medical Laboratory, Second Affiliated Hospital of Guangxi Medical University and Guangxi Huayin Medical Laboratory Co., Ltd. A commercial assay kit was employed for this procedure, in which the manufacturer had pre-optimized the target gene panel and primer sequences. For tNGS, sputum or viscous bronchoalveolar lavage fluid (BALF) samples were mixed with DTT liquefaction reagent in a 1.5 mL tube and centrifuged. After cell lysis, DNA was extracted from 500 μL of the homogenate using a BayBiopure Magnetic Pathogenic Microorganisms Nucleic Acid Kit (Guangzhou Bay Area Biotechnology Co., Ltd., China, Lot number: PMNM-LQ64-Magmix) or a Blood/Cell/Tissue Genomic DNA Extraction Kit (Shanghai Jiachu Biotechnology Co., Ltd., China, Lot number: YDP304-02), following the manufacturer’s protocols. After reverse transcription, the cDNA products were enriched by targeting specific regions with a PCR cycle set at 95°C for 3 minutes, followed by 23 cycles of 95°C for 25 seconds, 63°C for 120 seconds, and 72°C for 120 seconds, with a final extension at 72°C for 5 minutes, before being held at 4°C. Magnetic beads were used to purify the targeted PCR products. Then, 10 μL of the PCR product was used for secondary amplification and purified using magnetic beads once again. Sequencing of the purified libraries was performed using the MGISEQ-200RS High Throughput (Fast) Sequencing Kit (GI).

### Antibiotic resistant test

The identification and antimicrobial susceptibility testing (AST) of *P. aeruginosa* isolated from clinical specimens were performed using the Matrix-assisted laser desorption ionization time-of-flight mass spectrometry (MALDI-TOF MS) and the BD Phoenix M50 automatic microbial identification and drug susceptibility system. Susceptibility testing was done against the following antibiotics: ceftazidime, cefepime, meropenem, imipenem, piperacillin-tazobactam, aztreonam, tobramycin, ciprofloxacin, levofloxacin, ceftazidime-avibactam, cefoperazone-sulbactam and colistin. Antimicrobial sensitivity testing followed the guidelines established by the Clinical and Laboratory Standards Institute v33.0 (CLSI, 2023) and v34.0 (CLSI, 2024). The disk diffusion method was additionally employed for supplementary testing of relevant antibiotics.

Based on resistance patterns, *P. aeruginosa* was categorized into susceptible *P. aeruginosa* (SPA), MDR, and XDR groups (Magiorakos et al., 2012; Teo et al., 2021; Yaping Xu et al., 2017). In this study, MDR was defined as resistance to at least three classes of the following antibiotics: ceftazidime, cefepime, meropenem, imipenem, piperacillin-tazobactam, aztreonam, tobramycin, ciprofloxacin and levofloxacin. XDR was defined as resistance to all antibiotics except colistin. Additionally, *P. aeruginosa* was further classified into CRPA and carbapenem-sensitive *P. aeruginosa* (CSPA) (Teo et al., 2021).

### Statistical analysis

Data analysis was performed using SPSS software (IBM, USA), version 19.0. Enumeration data were expressed as cases/percentage [n (%)], and comparisons between groups were made via Chi-squared tests, Continuity-corrected Chi-square Test or Fisher’s exact test. Normally distributed continuous data were presented as mean ± standard deviation (SD). Comparisons between the two groups were conducted using independent samples t-tests. For nonnormally distributed continuous data, results were expressed as median (interquartile range), M (Q1, Q3), and the Wilcoxon rank-sum test was used for group comparisons. A *p*-value <0.05 was considered to indicate statistically significant test results.

## Results

### Patient characteristics

The clinical samples included 61 males and 44 females, aged between 7 to 86 years. Isolates were derived from BALF (n=91), sputum (n=5), blood cultures (n=2), cerebrospinal fluid (n=2), tissue (n=2), and other origins (n=3). 55 isolates were collected from the pneumology department, 33 from intensive care unit (ICU), 4 from the rehabilitation department, 3 from the orthopedics department, 3 from cardiovascular thoracic surgery, 2 from emergency ward observation, and 5 from other departments.

### Antimicrobial susceptibility profile of *P. aeruginosa*

**Figure 1:**
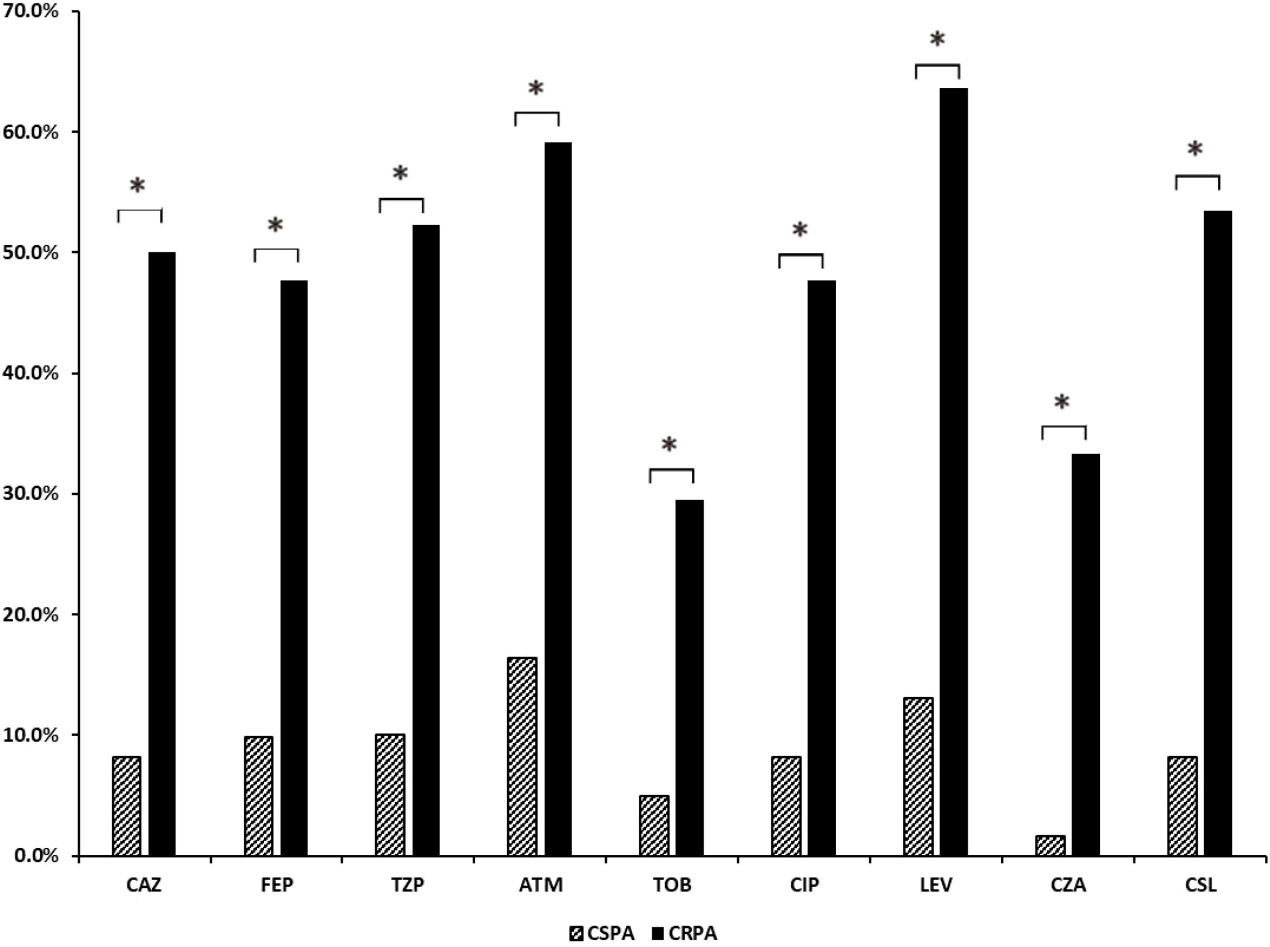
Analysis of drug resistance rates in CSPA and CRPA groups. CAZ, ceftazidime; FEP, cefepime; MEM, meropenem; IPM, imipenem; TZP, piperacillin-tazobactam; ATM, aztreonam; TOB, tobramycin; CIP, ciprofloxacin; LEV, levofloxacin; CZA, ceftazidime-avibactam; CSL, cefoperazone-sulbactam. *, The results indicate a statistically significant difference between the two groups.

In terms of drug resistance, 55 isolates were classified as SPA, 50 as MDR, and 6 as XDR. Among the MDR, 72% exhibited additional resistance to aztreonam, 56% to cefoperazone-sulbactam, 28% to tobramycin, and 30% to ceftazidime-avibactam. All isolates remained susceptible to colistin. Additionally, there were 61 cases of CSPA and 44 cases of CRPA, and among the CRPA isolates, resistance rate was significantly higher than that of CSPA (*p*<0.05) (**Figure 1**). All of these isolates were also susceptible to colistin.

### Analysis of virulence gene *exoY* and antimicrobial resistance

**Figure 2:**
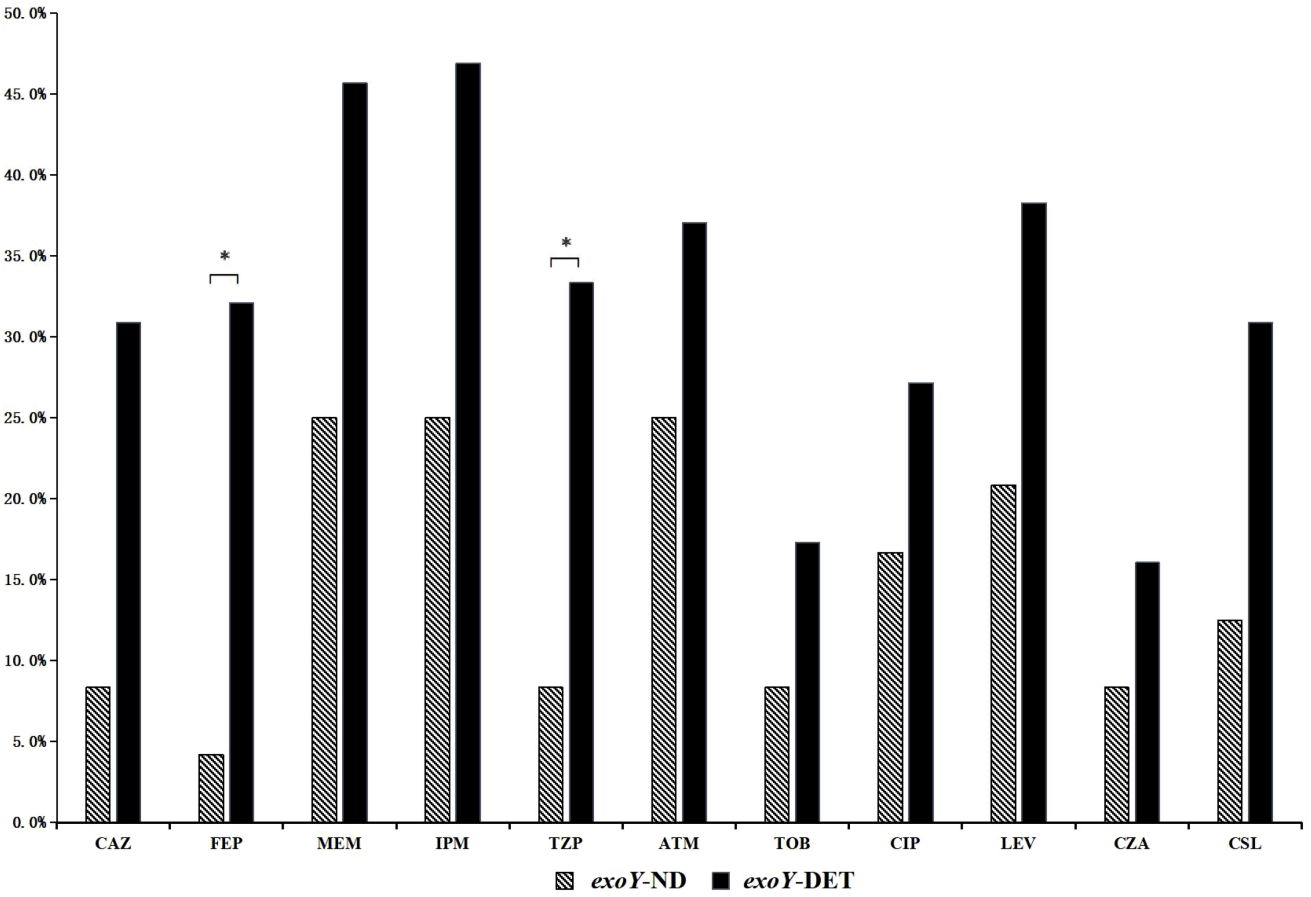
Resistance rates to common antibiotics among *exoY*-ND and *exoY*-DET strains. *, The results indicate a statistically significant difference between the two groups.

Among the 105 P*. aeruginosa* isolates, 77.1% (81/105) were *exoY* detected (*exoY*-DET), and 22.9% (24/105) were *exoY* undetected (*exoY*-ND). The resistance rates of *exoY*-DET strains to cefepime and piperacillin-tazobactam were significantly higher than those of the *exoY*-ND strains (*p*<0.05) (**Figure 2**). In terms of different resistance patterns, the proportion of *exoY*-DET strains in MDR isolates was significantly higher than in SPA isolates (*p*<0.05). However, there was no significant difference in the prevalence of *exoY* between CSPA and CRPA (**Table 2**).

**Table 2:** Percentages of different resistance patterns carrying *exoY* (%).

Pattern	*exoY*-ND (n=24)	*exoY*-DET (n=81)	χ^2^	*p* value
SPA	70.8	46.9	4.247	**0.039**
MDR	29.2	53.1		
CSPA	75.0	53.1	3.652	0.056
CRPA	25.0	46.9		

SPA, susceptible *P. aeruginosa*; MDR, multidrug-resistant *P. aeruginosa*; CSPA, carbapenem-sensitive *P. aeruginosa*; CRPA, carbapenem-resistant *P. aeruginosa*.

Bold numbers denote statistically significant results (p < 0.05).

### Associations between *exoY* and patient clinical characteristics

There were no significant differences between the *exoY*-ND group and the *exoY*-DET group in terms of age, history of aggressive treatment within the past year, antibiotic use before hospitalization (within 90 days), presence of underlying diseases, hospital-acquired infection, ICU admission, as well as pre-*P. aeruginosa* detection intervention including invasive procedures, catheterization, mechanical ventilation, duration of antibiotic use time≥14 days, the number of antibiotic use type ≥3 types, and metrics including hospitalization time and outcomes (**Table 3**).

**Table 3:** Clinical characteristics for *exoY*-ND and *exoY*-DET groups of *P. aeruginosa* infection.

Clinical characteristics	*exoY*-ND (n=24)	*exoY*-DET (n=81)	Z/t/*χ* ^2^	*p* value
Age (Years) [n (%)]				
<30	0 (0.0)	3 (3.7)	3.735	0.443
31-50	6 (25.0)	12 (14.8)		
51-60	9 (37.5)	23 (28.4)		
61-70	4 (16.7)	24 (29.6)		
71-90	5 (20.8)	19 (24.4)		
Data before hospitalization [n (%)]				
Aggressive treatment within 1 year	13 (54.2)	43 (53.1)	0.009	0.926
Antibiotic use before hospitalization	16 (66.7)	58 (71.6)	0.217	0.641
Presence of underlying diseases	22 (91.7)	77 (95.1)	0.017	0.898
Hospital-acquired infection	12 (50.0)	36 (44.4)	0.230	0.631
ICU admission	7 (29.2)	25 (30.9)	0.025	0.874
Prior to isolating *P. aeruginosa* [n (%)]				
Invasive procedure	16 (66.7)	47 (58.0)	0.576	0.448
Catheterization	12 (50.0)	38 (46.9)	0.071	0.790
Mechanical ventilation	10 (41.7)	28 (34.6)	0.404	0.525
Antibiotic use time≥14 days	13 (54.2)	55 (67.9)	1.53	0.216
Antibiotic use type ≥3 types	14 (58.3)	38 (46.9)	0.966	0.326
Hospitalization time [M (Q1, Q3), d]	14 (9,33.0)	15 (9,32.5)	-0.367	0.714
Outcomes [n (%)]				
Death	1 (4.2)	4 (4.9)	0.000	1.000
Improvement	22 (91.7)	71 (87.7)		

### Drug resistance genes and drug resistance

The resistance genes that could be detected in this study including β-lactamase, vancomycins, methicillins, aminoglycosides, macrolides, sulfonamides, quinolones, chloramphenicols, tetracyclines, polymyxins and efflux pump class. For aminoglycoside resistance genes, we designed primers for: *aac, ant, aph, armA* and *rmtB* (**Supplementary Table 1**). But our study only detected *aac(3’), aac(6’)*, *armA*, *cmlA, bla*_TEM_, *floR*, *bla*_GES_ and *tetC* genes in *P. aeruginosa*.

Among the 105 isolates, 7 were detected the *aac*(6’) and *aac*(3’) resistance gene, and these exhibited significantly higher resistance to tobramycin compared to isolates undetected the gene (*p*<0.05). No significant difference was observed in the detection rate of the *aac(6’)aac(3’)* across the SPA and MDR, or CSPA and CRPA groups (**Table 4**). 5 isolates detected the *armA* resistance gene, and these isolates demonstrated significantly higher resistance to tobramycin compared to the *armA*-ND group as well (*p*<0.05). The *armA* resistance gene was detected exclusively in MDR and CRPA isolates (**Table 4**), and only one *armA*-DET isolate was classified as XDR (data not shown). In addition, the resistance genes *cmlA*, *bla*_TEM_, *floR*, *bla*_GES_, and *tetC* were detected in 13, 3, 2, 1, and 1 isolate(s), respectively.

**Table 4:** Correlation between drug resistance genes and resistance.

Pattern	*aac(6’)aac(3’)*-DET (n=7)	*aac(6’)aac(3’)*-ND (n=98)	*χ* ^2^	*p* value	*armA*-DET (n=5)	*armA*-ND (n=100)	*χ* ^2^	*p* value
TOB R^a^ (%)	71.4	25.5	4.688	**0.030**	100.0	25.0	13.000	**0.000**
TOB S^b^ (%)	28.6	74.5			0.0	75.0		
SPA (%)	42.9	53.1	0.017	0.896	0.0	55.0	5.720	**0.017**
MDR (%)	57.1	46.9			100.0	45.0		
CSPA (%)	42.9	59.2	0.202	0.653	0.0	61.0	7.209	**0.007**
CRPA (%)	57.1	40.8			100.0	39.0		

a Tobramycin resistance.

b Tobramycin susceptible.

SPA, susceptible *P. aeruginosa*; MDR, multidrug-resistant *P. aeruginosa*; CSPA, carbapenem-sensitive *P. aeruginosa*; CRPA, carbapenem-resistant *P. aeruginosa*.

Bold numbers denote statistically significant results (p < 0.05).

### Correlation between different resistance phenotypes and patient clinical characteristics

There were no statistically significant differences between SPA and MDR or between CSPA and CRPA in terms of age, history of aggressive treatment within the past year, and underlying diseases (*p*>0.05). However, significant differences were observed between groups regarding the use of antibiotics prior to hospitalization (within 90 days), hospital-acquired infections, ICU admission, as well as pre-*P. aeruginosa* detection interventions including invasive procedures, catheterization, mechanical ventilation, duration of antibiotic use time≥14 days, and the number of antibiotic use type ≥3 types (*p*<0.05). Following the detection of *P. aeruginosa*, the duration of antibiotic use time≥14 days, and the number of antibiotic use type ≥3 types, and the length of hospital stay were also significantly different between groups (*p*<0.05). These findings suggest that the detection of MDR and CRPA strains is associated with prolonged antibiotic use, a greater diversity of antibiotics, and extended hospital stays (**Table 5**).

**Table 5:** Clinical characteristics of patients with *P. aeruginosa* infections with different resistance patterns.

Clinical characteristics	SPA (n=55)	MDR (n=50)	Z/t/*χ* ^2^	*p* value	CSPA (n=61)	CRPA (n=44)	Z/t/*χ* ^2^	*p* value
Age (Years) [n (%)]								
<30	2(3.6)	1(2.0)	5.509	0.281	1(1.6)	2(4.5)	5.404	0.248
31-50	12(21.8)	6(12.0)			14(23.0)	4(9.1)		
51-60	18(32.7)	14(28.0)			19(31.1)	13(29.5)		
61-70	10(18.2)	18(36.0)			13(21.3)	15(34.1)		
71-90	13(23.6)	11(22.0)			14(23.0)	10(22.7)		
Data before hospitalization [n (%)]								
Aggressive treatment within 1 year	26(47.3)	30(60.0)	1.705	0.192	28(45.9)	28(63.6)	3.230	0.072
Antibiotic use before hospitalization	33(60.0)	41(82.0)	6.092	**0.014**	38(62.3)	36(81.8)	4.682	**0.030**
Presence of underlying diseases	50(90.9)	49(98.0)	1.305	0.253	56(91.9)	43(97.7)	0.747	0.387
Hospital-acquired infection	17(30.9)	31(62.0)	10.202	**0.001**	17(27.9)	31(70.5)	18.680	**0.000**
ICU admission	10(18.2)	22(44.0)	8.239	**0.004**	7(11.5)	25(56.8)	24.804	**0.000**
Prior to isolating *P. aeruginosa* [n (%)]								
Invasive procedure	27(49.1)	36(72.0)	6.449	**0.011**	27(44.3)	36(81.8)	15.022	**0.000**
Catheterization	19(34.5)	31(62.0)	7.914	**0.005**	19(31.1)	31(70.5)	15.834	**0.000**
Mechanical ventilation	14(25.5)	24(48.0)	5.761	**0.016**	11(18.0)	27(61.4)	20.783	**0.000**
Antibiotic use time≥14 days	28(50.9)	40(80.0)	9.712	**0.002**	31(50.8)	37(84.1)	12.399	**0.000**
Antibiotic use type ≥3 types	18(32.7)	34(68.0)	13.035	**0.000**	18(29.5)	34(77.3)	23.329	**0.000**
After detecting *P. aeruginosa* [n (%)]								
Antibiotic use time≥14 days	23(41.8)	32(64.0)	5.166	**0.023**	24(39.3)	31(70.5)	9.919	**0.002**
Antibiotic use type ≥3 types	16(29.1)	28(56.0)	7.79	**0.005**	16(26.2)	28(63.6)	14.692	**0.000**
Hospitalization time [M (Q1, Q3), d]	12(8,23)	22.5(12.5,49)	-3.243	**0.001**	12(8,17.5)	26.0(14,52.5)	-3.776	**0.000**
Outcomes [n (%)]								
Death	2(3.6)	3(6.0)	0.035	0.851	2(3.3)	3(6.8)	0.229	0.632
Improvement	51(92.7)	42(84.0)			57(93.4)	36(81.8)		

SPA, susceptible *P. aeruginosa*; MDR, multidrug-resistant *P. aeruginosa*; CSPA, carbapenem-sensitive *P. aeruginosa*; CRPA, carbapenem-resistant *P. aeruginosa*.

Bold numbers denote statistically significant results (p < 0.05).

### Comparative analysis of tNGS and whole genome sequencing results

As a newly developed platform for detecting virulence and resistance genes, the validity of tNGS needs to be confirmed. To address this issue, we conducted whole genome sequencing (WGS) on 10 enriched bacterial samples from the included studies and compared the results with those from the tNGS (**Supplementary Tables 1**, **2**). The results demonstrated a 100% concordance rate between WGS and tNGS for *exoY, cmlA, and armA*. However, although WGS achieved 100% detection rates for *mucA, fliC/D, flgC/D/E/G/H/I/J, flhB, fliI/J/M/P/Q/R, fleN*, and *aph(3’)-IIb*, 20% for *wzz* and 10% for *ant(2’’)*, tNGS failed to detect these genes. It should be noted that both *aac(6’)* and *wzy* were detected in Case 9 by WGS but were missed by tNGS, suggesting potential false negatives in tNGS (**Table 6**). These findings suggest that WGS outperforms tNGS in detecting virulence and resistance genes. Moreover, among 4,206 historical tNGS reports, *aph(3’)-IIb* was detected in 108 cases, mainly associated with *Staphylococcus aureus*. This observation implies that the tNGS primers targeting the *aph* resistance gene may have strain specificity and thus may not be suitable for detecting in *P. aeruginosa*.

**Table 6:** Comparative detection of virulence and resistance genes by WGS and tNGS in clinical samples.

Patients	Virulence genes		Resistance genes	
	WGS	tNGS	WGS	tNGS
Case 1	***mucA, fliC/D, flgC/D/E/G/H/I/J, flhB, fliI/J/M/P/Q/R, fleN*,** exoY	*exoY*	***aph(3’)-IIb***	*/*
Case 2	***mucA, fliC/D, flgC/D/E/G/H/I/J, flhB, fliI/J/M/P/Q/R, fleN*,** exoY	*exoY*	***aph(3’)-IIb***	*/*
Case 3	***mucA, fliC/D, flgC/D/E/G/H/I/J, flhB, fliI/J/M/P/Q/R, fleN*,** exoY	*exoY*	***aph(3’)-IIb***	*/*
Case 4	***mucA, fliC/D, flgC/D/E/G/H/I/J, flhB, fliI/J/M/P/Q/R, fleN***	*/*	***aph(3’)-IIb***	*/*
Case 5	***mucA, fliC/D, flgC/D/E/G/H/I/J, flhB, fliI/J/M/P/Q/R, fleN*,** exoY	*exoY*	***aph(3’)-IIb*,** cmlA	*cmlA*
Case 6	***mucA, fliC/D, flgC/D/E/G/H/I/J, flhB, fliI/J/M/P/Q/R, fleN*,** exoY	*exoY*	***aph(3’)-IIb***	*/*
Case 7	***mucA, fliC/D, flgC/D/E/G/H/I/J, flhB, fliI/J/M/P/Q/R, fleN***	*/*	***aph(3’)-IIb***	*/*
Case 8	***mucA, fliC/D, flgC/D/E/G/H/I/J, flhB, fliI/J/M/P/Q/R, fleN***	*/*	***aph(3’)-IIb***	*/*
Case 9	***mucA, fliC/D, flgC/D/E/G/H/I/J, flhB, fliI/J/M/P/Q/R, fleN, wzz*, wzy,** exoY	*exoY*	***aph(3’)-IIb, aac(6’), ant(2’’)*,** cmlA, armA	*cmlA, armA*
Case 10	***mucA, fliC/D, flgC/D/E/G/H/I/J, flhB, fliI/J/M/P/Q/R, fleN, wzz*,** exoY, wzy	*exoY, wzy*	***aph(3’)-IIb***	*/*

WGS, whole genome sequencing; tNGS, targeted next-generation sequencing.

Boldface entries highlight discordant findings between whole genome sequencing (WGS) and targeted next-generation sequencing (tNGS).

## Discussion

The infection potential of *P. aeruginosa* is partly attributable to its virulence factors and partly to its ability to metabolize a wide range of antibiotics, which collectively enhance its infectivity potential (Botelho et al., 2019), and it is crucial to understand the genetic mechanisms that underpin both of these sources of drug resistance. tNGS has the advantage of enriching genetic targets for specific pathogens or pathogen groups as well as other genes of interest (Gaston et al., 2022; Ma et al., 2024). Due to its high sensitivity, affordability, and strong concordance rate, tNGS has begun to emerge as a valuable tool in the clinical detection of infectious diseases (Liu et al., 2024). In this study, a commercial assay kit was used where genes and primers tested were preselected by the manufacturer. By December 20, 2024, a total of 4,206 specimens had been tested using tNGS. Among these, respiratory samples accounted for 60.0%, and blood samples accounted for 32.9%. Non-sterile body fluid samples frequently contain mixed microbes, including diverse Gram-negative and Gram-positive bacteria, viruses, and other pathogens, resulting in complex detected resistance and virulence genes. To minimize interference from resistance and virulence genes of other bacteria, we exclusively selected samples in which Gram-negative bacteria consisted solely of *P. aeruginosa* and for which both microbial culture and AST data were available.

Of the 105 cases, the sequence count for *P. aeruginosa* totaling 92,857(55,184-122,118), and the minimum sequence count was 949. This suggests that when the sequence count for *P. aeruginosa* is below a certain threshold, its colony count in culture may be low or even undetectable. Since culture is still the gold standard for bacterial confirmation, clinicians should carefully consider this diagnostic limitation when making antibiotic treatment strategies. And detecting substantial amounts of *P. aeruginosa* in respiratory tract specimens doesn’t mean an infection, as this opportunistic pathogen may colonize alongside polymicrobial flora. In such cases, the hospital’s microbial staff, after reviewing both culture and smear results, considered it may belong to typical pharyngeal flora and ultimately did not conduct drug susceptibility testing, or the sample was deemed unqualified and required additional inspection.

Virulence gene selection criteria prioritize clinical relevance, functional representativeness and detection feasibility. For clinical relevance, genes should be linked to infection severity or therapeutic decisions. For example: Based on clinical phenotype correlations and detection positivity rates, *exoY*-DET strains demonstrate higher prevalence in burn, bacteremia, urine, and respiratory samples (51.7-95.0%), whereas *exoT* exhibits substantial inter-study variability in detection rates (5-100%). Although *exoU* and *exoS* exhibit potent virulence, their limited carriage rates (approximately 13.3-64.5% and 20.0-38.46%, respectively) and poor representativeness across clinical cohorts restrict their broader applicability (Azimi et al., 2016; Jabalameli et al., 2012; Ullah et al., 2023). Regarding biofilm-related genes, the *mucA* mutation directly induces alginate overproduction, which is strongly associated with chronic pulmonary infections and mucoid colony formation in cystic fibrosis (CF) patients (Schofield et al., 2021). In contrast, Psl and Pel, while contributing to biofilm formation, are primarily linked to acute-phase infections or environmental adaptation, lacking well-defined clinical biomarkers (de Sousa et al., 2021). For functional representativeness, the Wzy/Wzz system regulates the synthesis of the O-antigen of lipopolysaccharide (LPS), determining the serotype of *P. aeruginosa* (e.g., O1–O20). This regulation impacts host immune recognition and antibody neutralization efficiency (Islam et al., 2010). Meanwhile, type IV pili (encoded by *pilA, pilB, pilC, pilQ*) are involved in adhesion and motility, but their functions can be partially compensated by flagella or other adhesins (e.g., CupA) (de Sousa et al., 2021).

In this study, the virulence factors were classified into nine functional categories: toxins, adhesion and invasion systems, secretion systems, biofilm and polysaccharide production, iron uptake mechanisms, LPS biosynthesis, genotoxins, flagella and motility, and heme uptake and utilization systems (**Supplementary Table 2**). Among them, the virulence genes specific to *P. aeruginosa* include *exoY* and *mucA*, while those shared with other bacteria comprise *wzy, wzz, lpxA/B/C/D/L/M, fleN, flgC/D/E/G/H/I/J*, and *fliC/D/I/J/M/P/Q/R*. However, only the *exoY* and *wzy* genes were detected in this study. Regarding resistance genes, only *aac(6’)*, *aac(3’)*, *armA*, *cmlA*, *bla*_TEM_, *floR*, *bla*_GES_, and *tetC* were detected.

ExoY is one of the effectors injected by the T3SS of *P. aeruginosa* into host cells. Inside eukaryotic cells, ExoY interacts with F-actin, which stimulates its potent nucleotidyl cyclase activity to produce cyclic nucleotide monophosphates (cNMPs), which disrupts the actin cytoskeleton and increases endothelial permeability (Galle et al., 2012; Mancl et al., 2020; Reynolds and Kollef, 2021; Silistre et al., 2021). Among the 105 P*. aeruginosa* isolates in this study, 81 (77.1%) were detected the *exoY* virulence gene, which is inconsistent with previous findings (Azimi et al., 2016; Ullah et al., 2023), possibly due to variations in specimen types. This study primarily used respiratory tract specimens, whereas other studies have focused on wound or secretion samples. The resistance rate of *exoY*-DET strains to testing drugs was higher than that of *exoY*-ND strains, though with statistically significant differences observed only for cefepime and piperacillin-tazobactam (*p*<0.05). Additionally, the proportion of *exoY*-DET strains in the MDR group was significantly higher than that in SPA isolates (*p*<0.05). These findings suggest that *P. aeruginosa* isolates that carry the *exoY* gene exhibit a more serious degree of drug resistance.

No significant differences were observed between the *exoY*-DET and *exoY*-ND groups in clinical features such as the duration and type of antibiotics used, hospitalization time and outcome. This is possibly because ExoY counteracts the overall cytotoxicity of *P. aeruginosa* toward human epithelial cells and may exert a protective role at certain stages of bacterial infection, potentially facilitating host colonization or contributing to the establishment and/or maintenance of chronic infection (Silistre et al., 2021). This tNGS panel balances the detection of antimicrobial resistance and virulence genes across multiple bacterial pathogens (**Supplementary Table 1, 2**), which limits the proportion of *P. aeruginosa*-specific gene targets. However, *exoS* or *exoU* may hold greater direct diagnostic relevance when prioritizing strongly virulent factors. Future tNGS protocols could incorporate multiplex detection of *exoS, exoU, and exoT* alongside integrated analysis of *exoY* with other T3SS effector proteins to comprehensively evaluate their synergistic contributions in polymicrobial infections.

Biosynthesis of B-band LPS in *P. aeruginosa* follows the Wzy-dependent pathway, which requires the integral inner membrane proteins Wzx O-antigen [O-Ag] flippase), Wzy (O-Ag polymerase), and WaaL (O-Ag ligase), the O-Ag chain length is determined by Wzz, a polysaccharide copolymerase type 1 protein (Islam et al., 2010). Many important bacterial virulence factors are encoded by prophage or have a phage origin. Due to this, Wzy may greatly contribute to *P. aeruginosa* virulence (Kaluzny et al., 2007). However, no significant differences were observed in drug sensitivity or clinical characteristics between the *wzy*-DET and *wzy*-ND groups (**Supplementary Table 3**, **Supplementary Figure 1**).

The *lpx* series is involved in lipid A biosynthesis and is conserved across Gram-negative bacteria. Currently, the best-characterized lipid A synthesis pathway is the Raetz pathway in *Escherichia coli* (Romano and Hung, 2023). Regarding flagellar assembly, the *fleN, flgC/D/E/G/H/I/J*, and *fliC/D/I/J/M/P/Q/R* genes are widely distributed across Gram-negative bacteria, including *P. aeruginosa*, *Salmonella enterica*, *Yersinia* spp., and *Shigella* spp (Erhardt et al., 2010; Oladosu et al., 2024). However, none of these genes were detected in our current study.

As a highly adaptable opportunistic pathogen, *P. aeruginosa* relies on the synergistic effects of multifaceted virulence mechanisms to establish infections. Beyond the well-characterized T3SS and LPS, it secretes a variety of exotoxins and proteases to directly disrupt host tissues. For example, Elastase (LasB, LasA) specifically degrade elastin-rich connective tissues in pulmonary structures (Casilag et al., 2016), while phospholipases and lipases disrupt surfactant function and modulate local immune responses (Korotkov et al., 2012; Ostroff et al., 1990). Exotoxin A inhibits protein synthesis by ADP-ribosylation of cell elongation factor-2 in the host cell, consequently leading to cell death (Allured et al., 1986). Further proteolytic activity is demonstrated by PrpL, a serine protease targeting multiple host substrates including casein, lactoferrin, transferrin, elastase, and decorin (Wilderman et al., 2001). Simultaneously, biosurfactant molecules like rhamnolipids serve dual functions, facilitating biofilm maturation while impairing host immune cell function (Alhede et al., 2009; Pearson et al., 1997).

The pathogen’s iron acquisition systems constitute another critical virulence dimension. *P. aeruginosa* employs high-affinity siderophores (pyoverdine and pyochelin) and direct heme-iron uptake via PhuR/HasR transporters to overcome iron-deficient environment (Ochsner et al., 2000; Schalk and Guillon, 2013). This efficient iron-capturing ability not only supports bacterial proliferation but also modulates virulence gene expression through iron-responsive regulatory networks (Minandri et al., 2016). Chronic infection strategies center on sophisticated biofilm development mediated by exopolysaccharides (Psl, Pel, and alginate) that establish protective matrices, and dynamically regulates virulence factor expression through quorum-sensing (QS) systems (Las, Rhl) (de Sousa et al., 2021). Moreover, the type V secretion system (T5SS) strengthens its ecological niche competitiveness by promoting biofilm formation and auto-aggregation, and influencing the host’s response to bacterial infection (Borlee et al., 2010; Salacha et al., 2010). The interplay of these virulence mechanisms poses significant clinical challenges. Future tNGS design could consider incorporating more virulence factor detection.

AAC enzymes are a subset of aminoglycoside-modifying enzymes. Acetyltransferases, in particular, play a crucial role in bacterial resistance due to their ability to inactivate several medically important aminoglycosides, including gentamicin, tobramycin and amikacin (Dolgusevs et al., 2024; Schwocho et al., 1995). In this study, the *aac(6’)aac(3’)* resistance gene was detected in 7 of 105 P*. aeruginosa* isolates, a detected rate of 6.7%. The resistance rate to tobramycin among *aac(6’)aac(3’)*-DET strains was 71.4% (5/7), which was significantly higher than that of *aac(6’)aac(3’)*-ND strains (*p*<0.05) (**Table 3**). This suggests that the presence of *aac(6’)aac(3’)* were highly correlated with tobramycin resistance.

16S RMTases represent one of the most concerning mechanisms of resistance to aminoglycosides. Among the G1405 16S RMTases, ArmA and AmtB are the most widely distributed, having been found in various species of *Enterobacteriaceae*, as well as in *P. aeruginosa* and *Acinetobacter baumannii* (O’Hara et al., 2013). In this study, *armA* resistance genes were detected in 5 cases, and the tobramycin resistance rate was 100% in the *armA*-DET group, significantly higher than that in the *armA*-ND group (*p*<0.05). *armA* was present only in MDR and CRPA strains, and the degree of drug resistance was severe. In addition to colistin, only penicillins with b-lactamase inhibitors, ceftazidime, ceftazidime-avibactam, cefoperazone-sulbactam and/or aztreonam remained sensitive. Strains detected both the *aac(6’)aac(3’)* and *armA* resistance genes exhibited higher resistance to aminoglycosides; however, 68.7% of tobramycin-resistant strains did not detect either the *aac(6’)aac(3’)* or *armA* genes. *cmlA* was associated with chloramphenicol resistance, and *P. aeruginosa* is naturally resistant to chloramphenicol (Osei Sekyere and Reta, 2020), but only 12.4% percent of *P. aeruginosa* had the gene detected. Additionally, resistance genes for β-lactamase, chloramphenicol, and tetracyclines were detected in a subset of samples. These findings raise concerns about the reliability of the use of genomic diagnostics as the sole tool for resistance prediction, as dependence on genomics alone may lead to erroneous diagnoses and inappropriate therapeutic decisions (Sommer et al., 2020).

tNGS and drug susceptibility tests were repeated in six patients within six months. One patient who was diagnosed with severe pneumonia caused by *P. aeruginosa* was admitted to the ICU and the infection could not be controlled. After six days, a second BALF was collected for tNGS analysis. The results revealed that *P. aeruginosa* had detected the *exoY*, *armA* and *cmlA* genes. The abrupt detection of *exoY*, *armA* and *cmlA* genes in *P. aeruginosa* may reflect a polymicrobial infection with population dynamics. Prolonged antimicrobial selection drives resistant strain dominance through competitive advantage, achieving tNGS-detectable thresholds. As a result, the drug susceptibility profile shifted from full sensitivity to multiple drug resistance, with susceptibility remaining only to cefoperazone-sulbactam and colistin. Unfortunately, treatment ultimately failed. This case highlights the utility of tNGS not only in identifying the primary pathogens of infection but also in predicting changes in drug resistance through the detection of resistance and virulence genes. Such early identification of resistance mechanisms can guide timely adjustments in antimicrobial therapy, even before traditional drug susceptibility results are available. But the mechanisms of resistance to carbapenems or other drugs among *P. aeruginosa* strains are multifactorial, such as the fact that repression or inactivation of the carbapenem porin OprD and the hyperexpression of the chromosomal cephalosporinase AmpC are associated with reduced susceptibility to carbapenems (Hu et al., 2021). This suggests that, in addition to genetic factors, other mechanisms may contribute to resistance.

In another case, a patient infected with *P. aeruginosa* did not detected virulence or drug resistance genes, and the patient’s symptoms failed to improve after five days of treatment. On the sixth day, a follow-up test revealed that the pathogen was still *P. aeruginosa*, and it still did not detect any virulence or resistance genes. However, the strain had developed MDR, with only carbapenems, tobramycin, and ceftazidime-avibactam remaining effective. A genomic study of 40 nosocomial *P. aeruginosa* strains demonstrated the organism’s dynamic and highly plastic genome, which enables clinical strains to acquire resistance. These adaptive mechanisms allow *P. aeruginosa* to respond to selective pressures quickly, such as those from prolonged antibiotic treatment (Liu et al., 2022; Ventero et al., 2023). Therefore, continuous monitoring of *P. aeruginosa* drug susceptibility is essential, even in the absence of gene acquisition.

In addition, drug sensitivity was continuously monitored in 41 cases, and significant changes were observed in 5. Among these, 2 cases transitioned from full sensitivity to MDR combined with carbapenem resistance, 2 developed into CRPA, and 1 became MDR. However, 10 patients with *P. aeruginosa* showed no change in their drug sensitivity profiles, and no significant differences in drug use were noted. This suggests that in addition to antibiotic use, the establishment of an infection probably depends much more on adaptation to a range of other selective factors within the human host such as the immune system, altered nutrient availability, fluctuating oxygen concentrations, and the composition of the indigenous microbiota (La Rosa et al., 2018; Pickard et al., 2017; Sommer et al., 2020).

Between the SPA and MDR groups, as well as between the CSPA and CRPA groups, significant differences were observed in several factors, including the use of antibiotics before hospitalization (within 90 days), hospital-acquired infections, ICU admission, as well as pre-*P. aeruginosa* detection interventions including invasive procedures, catheterization, mechanical ventilation, duration of antibiotic use time≥14 days, and the number of antibiotic use type ≥3 types (*p*<0.05). After the detection of *P. aeruginosa*, the duration of antibiotic use time≥14 days, and the number of antibiotic use type ≥3 types, and the length of hospital stay were also significantly different (*p*<0.05). Furthermore, the detection of MDR and CRPA strains appears to lead to an increase in the duration and variety of antibiotic use, as well as an extended length of hospital stay.

Finally, carbapenem resistance in *P. aeruginosa* can complicate treatment regimens, given how often *P. aeruginosa* is resistant to other antimicrobials. In our study, 41.9% of *P. aeruginosa* isolates were found to be carbapenem-resistant. Notably, over 81.8% of the patients with carbapenem-resistant isolates had prior healthcare exposures, highlighting the significance of nosocomial infections in *P. aeruginosa* infections. However, these results may be subject to bias, as patients who underwent tNGS testing were primarily from the ICU and respiratory departments, where clinical situations are typically more severe. Additionally, after prolonged treatment, *P. aeruginosa* is more likely to develop acquired resistance, which may further contribute to the severity of an infection.

tNGS workflows enrich specific genetic targets for sequencing and therefore have the advantage of enriching genetic targets for specific pathogens or pathogen groups. Nevertheless, our analysis revealed that certain virulence and antimicrobial resistance genes identified through WGS were undetectable within the tNGS analytical pipeline. Actually, multiplex PCR scaling to large panels for broad range of most common pathogens causes the nonlinear increase of primer dimer species that reduces tNGS mapping rates (Liu et al., 2024).

A limitation of this study stems from the lack of scientifically substantiated rationale underlying the manufacturer-curated virulence gene panel selection, with future studies benefiting from the inclusion of clinically relevant virulence determinants through expanded genomic profiling.

## Conclusions

tNGS is a valuable tool for detecting major pathogens and providing information on virulence and resistance genes. But present detection systems cover only a subset of known virulence/resistance genes, systematic incorporation of newly characterized, medically relevant genes into pipelines warrants urgent development. Although drug resistance genes correlate with resistance phenotypes, they cannot fully replace traditional drug susceptibility tests. A comprehensive approach that integrates both genomic and phenotypic data is thus essential for accurate clinical decision-making.

## Data Availability

The original contributions presented in the study are included in the article/**Supplementary Material**. Further inquiries can be directed to the corresponding author.
