# Supplementary material for: The relationship between virulence and drug resistance genes in Pseudomonas aeruginosa and antibiotic resistance: a targeted next-generation sequencing approach
Source: Front Cell Infect Microbiol. 2025 May 26;15:1563741. doi: 10.3389/fcimb.2025.1563741 (PMC12146319; doi:10.3389/fcimb.2025.1563741)

**Supplementary Table 1.** Detection list of resistance genes

| **Classification** | **Name** |
| --- | --- |
| β-lactamase | *bla*_KPC_, *bla*_SME_, *bla*_IMI_, *bla*_GES_, *bla*_NDM_, *bla*_IMP_, *bla*_VIM_, *bla*_OXA (1, 2, 9, 23, 24, 48, 51, 55, 69, 181, 198),_ *bla*_PER_, *bla*_SHV_, *bla*_TEM_, *bla*_VEB_, *bla*_ACC_, *bla*_ACT_, *bla*_ADC_, *bla*_CMY_, *bla*_DHA_, *bla*_MIR,_ *ampC* |
| Vancomycins | *vanA, vanB, vanC, vanD, vanE, vanG* |
| Methicillins | *mecA, mecB, mecC* |
| Aminoglycosides | *aac(2'), aac(3), aac(6'), ant, aph, armA, rmtB* |
| Macrolides | *ereA, ereB, ermB, mefA, mefE, msrA, macA, mphB* |
| Sulfonamides | *dfr, sul* |
| Quinolones | *qnrA* |
| Chloramphenicols | *cat, cmlA, floR* |
| Tetracyclines | *tetB, tetC, tetD, tetE, tetK, tetL, tetM, tetO, tetQ, tetS, tetT, tetW* |
| Polymyxins | *mcr-1* |
| Efflux pump class | *acr, adeF, adeG, tolC* |

**Supplementary Table 2.** Detection list of virulence genes

| Functional Category | Representative Genes |
| --- | --- |
| Toxins | *stx1A/B, stx2A/B, cdtA/B/C, hlyA/B/C/D, cnf1, eltA/B, estIa, east1, pet, pic, senB* |
| Adhesion and Invasion | *ompA, csuB-E, ibeA/B/C, set1A/B,* |
| Secretion Systems | *exoY* |
| Biofilm and Polysaccharide | *wzy, wzz, kpsD/M/T, pgaA-D, mucA* |
| Iron Uptake | *iucD, entA/C/D/F, iroB/D/E/N, ybt* series*, all* series*, irp2, fepD* |
| LPS Biosynthesis | *lpx* series*, lpsB, gtrA/B/II* |
| Genotoxins | *clbA-clbS* |
| Flagella and Motility | *fliC/D, flgC/D/E/G/H/I/J, flhB, fliI/J/M/P/Q/R, fleN* |
| Heme Uptake and Utilization Systems | *chuA, chuS, chuT, chuU, chuW, chuY* |

**Supplementary Table 3.** Clinical characteristics for *wzy*-DET and *wzy*-ND groups of *P. aeruginosa* infection

| **Clinical characteristics** | ***wzy*-DET（n=13）** | ***wzy*-ND（n=92）** | **Z/t/χ2** | ***p* value** |
| --- | --- | --- | --- | --- |
| Age (Years) [n (%)] |  |  |  |  |
| <30 | 0 | 3 | 1.802 | 0.772 |
| 31-50 | 2 | 16 |  |  |
| 51-60 | 3 | 29 |  |  |
| 61-70 | 5 | 23 |  |  |
| 71-90 | 3 | 21 |  |  |
| Data before hospitalization [n (%)] |  |  |  |  |
| Aggressive treatment within 1 year | 8 | 48 | 0.401 | 0.526 |
| Antibiotic use before hospitalization | 11 | 63 | 0.755 | 0.385 |
| Presence of underlying diseases | 13 | 86 | 1.637 | 0.201 |
| Hospital-acquired infection | 8 | 40 | 1.497 | 0.221 |
| ICU admission | 5 | 27 | 0.447 | 0.502 |
| Prior to isolating *P. aeruginosa* [n (%)] |  |  |  |  |
| Invasive procedure | 10 | 53 | 1.057 | 0.304 |
| Cannulation | 9 | 41 | 1.877 | 0.771 |
| Mechanical ventilation | 4 | 34 | 0.016 | 0.900 |
| Antibiotic used ≥ 14 days | 10 | 58 | 0.450 | 0.503 |
| Antibiotic classes used ≥3 types | 9 | 43 | 1.493 | 0.222 |
| Hospitalization time [M (Q1, Q3), d] | 14(9,33.0) | 15(9,32.5) | -0.589 | 0.556 |
| Outcomes [n (%)] |  |  |  |  |
| Death | 0 | 5 | 0.049 | 0.825 |
| Improvement | 13 | 80 |  |  |

**Supplementary Figure 1.**  Resistance rates to common antibiotics among *wzy*-ND and *wzy*-DET strains.


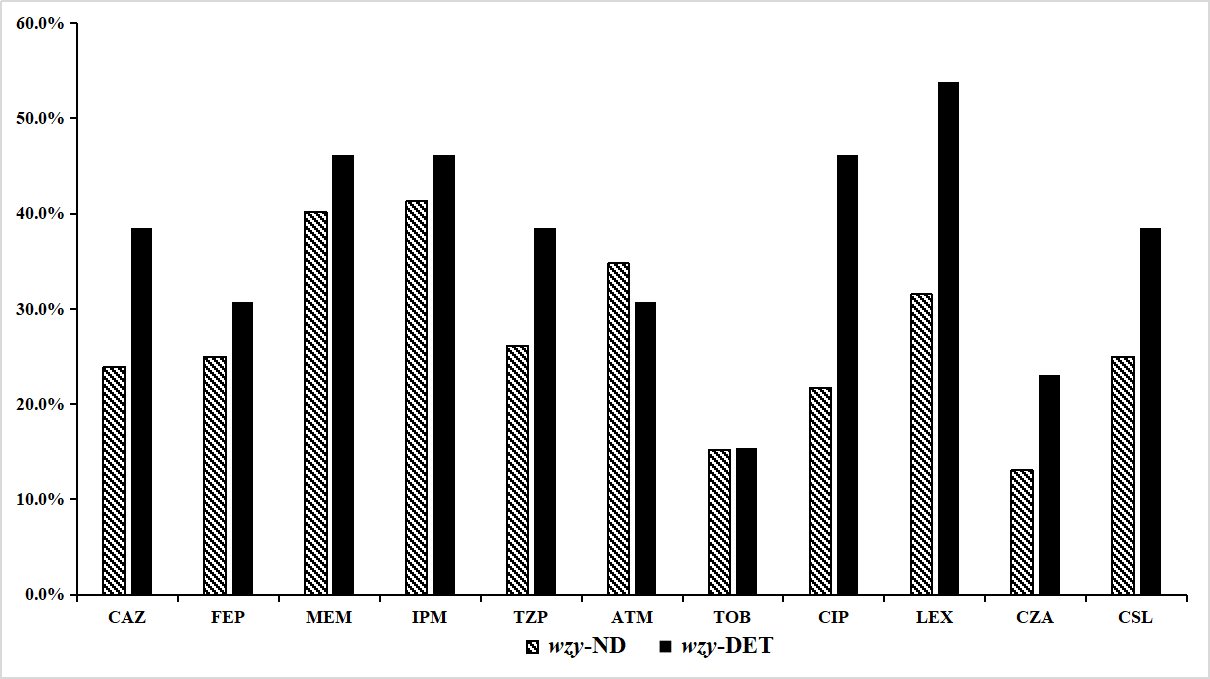

Supplement: Supplementary file 1 [file Table1.docx]
